# Incomplete Gastric Obstruction Due to Herniation of Stomach Into Parastomal Hernia: A Case Report

**DOI:** 10.7759/cureus.22479

**Published:** 2022-02-22

**Authors:** Prokopis Christodoulou, Stavros-Chrysovalantis Liapis

**Affiliations:** 1 Surgical Department, General Hospital of Volos, Volos, GRC

**Keywords:** stomach herniation, end colostomy, gastric outlet obstruction, stoma related complications, parastomal hernia

## Abstract

Parastomal hernia is the most common complication after surgical procedures that lead to the creation of a stoma. Most commonly in the hernia sac, there is omentum or small intestine or colon. The presence of the stomach as hernia’s content is a rare event. Herein, we present the case of a 68-year-old woman, who was hospitalized in our surgical unit with incomplete gastric obstruction due to herniation of the stomach into a parastomal hernia and who was managed conservatively.

## Introduction

A parastomal hernia considers an incisional hernia and is a common complication that occurs after surgeries that lead to the formation of a stoma, either colostomy or ileostomy [1]. According to the European Hernia Society, one-third of the patients with a stoma will develop a parastomal hernia in the first 12 months, while in more than half, the hernia will appear in a long-term period [2]. Most commonly, the hernia sac contains omentum or small intestine or colon [3]. The presence of stomach in the hernia sac is rare, and there are few reports in the literature.

## Case presentation

**Figure 1:**
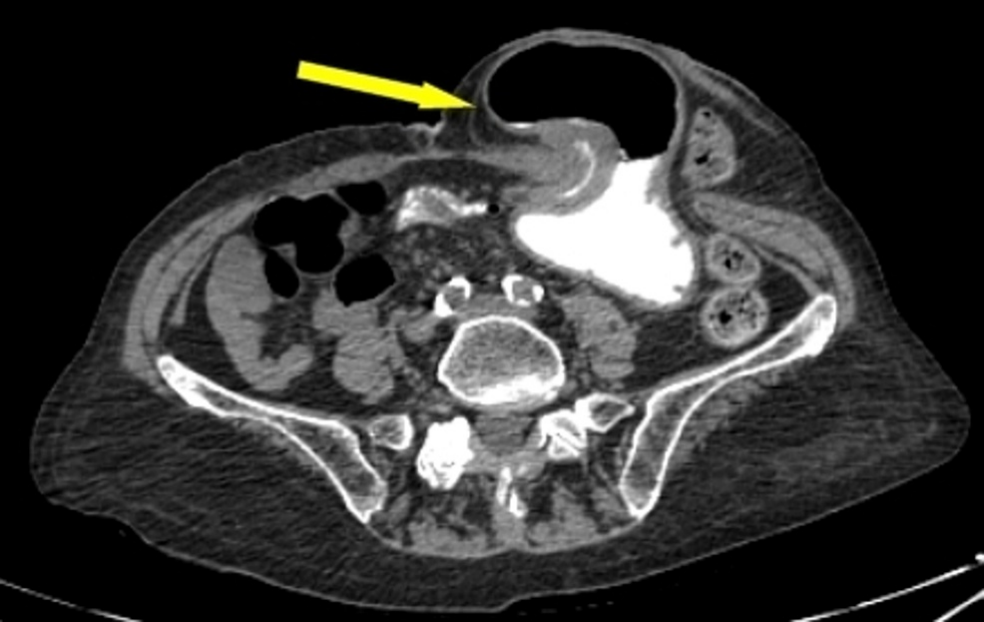
Axial view of abdominal CT scan, yellow arrow indicates the presence of stomach into parastomal hernia.

**Figure 2:**
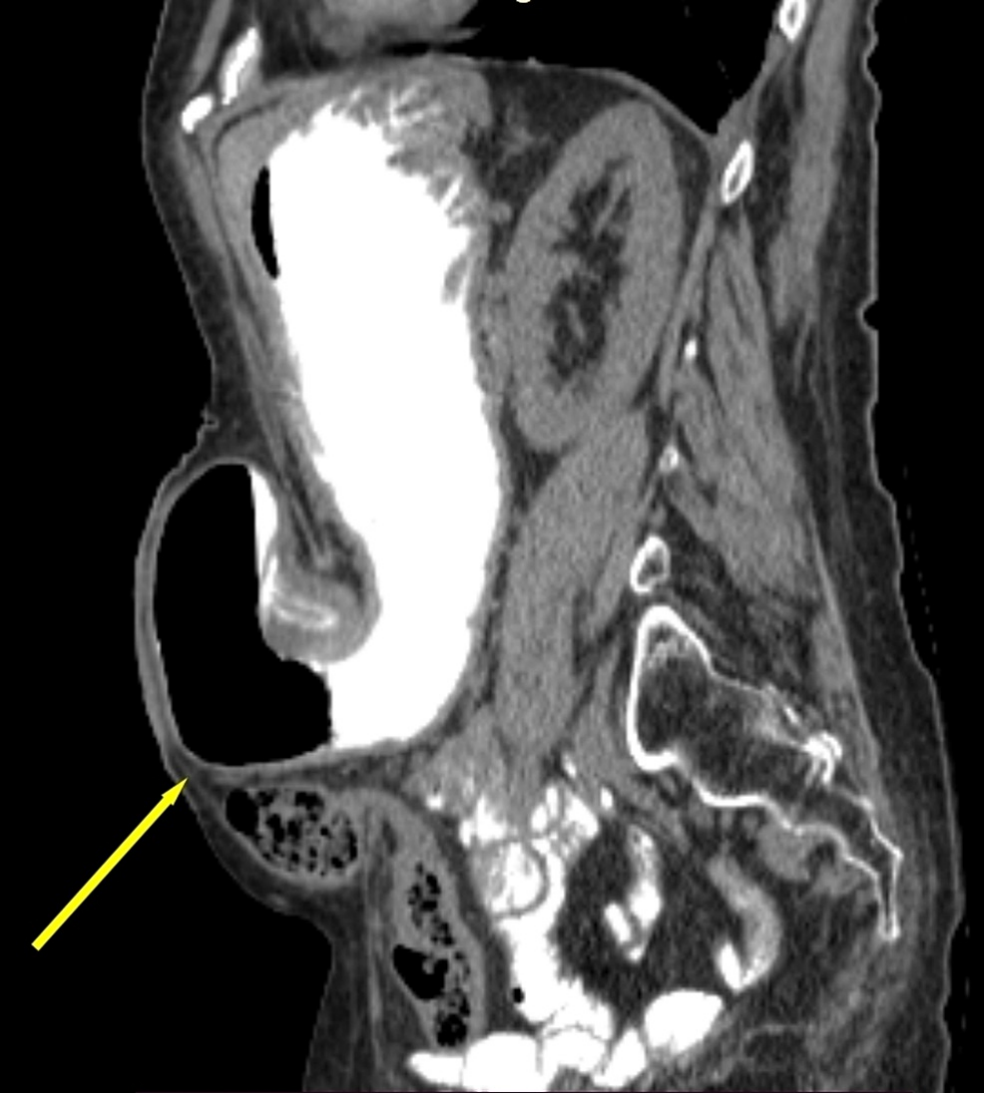
Sagittal view in abdominal CT scan, yellow arrow indicates stomach herniation.

A 68-year-old female was admitted to the emergency department due to multiple episodes of vomiting, nausea, and mild abdominal pain, which were started four hours before. Her medical history included hysterectomy with bilateral salpingo-oophorectomy due to malignancy and simultaneous Hartmann’s procedure because of local extension of tumor two years ago, small-cell lung cancer under chemotherapy that was diagnosed three months ago, and smoking. Upon arrival, her vital signs were stable. The physical examination revealed mild pain in the palpation of the left lumbar region, mild meteorism, and slightly reduced bowel sounds. Electrocardiogram (ECG) and blood tests were performed at an initial stage and were normal. A nasogastric tube was placed on the patient, and gastrografin was administered through it. The patient underwent an abdominal CT scan, which revealed incomplete gastric outlet obstruction caused by herniation of the stomach in a parastomal hernia (Figures 1, 2).

The patient was hospitalized in our surgical unit, and she was managed conservatively with intravenous liquids and bowel rest. The nasogastric tube was removed on the second day of hospitalization, and the patient started oral feeding. The patient who was discharged after 72 hours clinically improved with good tolerance of oral feeding and normal defecation. We suggested the patient an elective surgical restoration of hernia; however, she refused due to personal reasons.

## Discussion

There is no agreement regarding the incidence of parastomal hernia as the range of it varies and reaches up to 52% [4]. Stomach as the content of parastomal hernia has few reports in the literature, and according to a systematic review, only 12 cases of it have been described until now [5]. Important risk factors that increase the possibility of developing a parastomal hernia are obesity, female gender, age greater than 60 years old, chronic pulmonary disease, presence of malignancy, and the type of stoma [6,7]. Regarding the type of stoma, among end or loop ileostomy and end or loop colostomy, end colostomy is associated with higher probabilities of developing a parastomal hernia [8]. As we noticed in this case, our patient had several factors to develop a parastomal hernia including gender, age, type of stoma, and the presence of respiratory malignancy. Most of the patients with stomach herniation present with gastric outlet obstruction either completely or incompletely, or they have a more rambunctious clinical image due to incarcerated and perforated stomach [9]. Diagnosis includes high clinical suspicion along with abdominal CT scan findings [10]. To establish a common language among doctors, the European Hernia Society suggested a classification of parastomal hernia that includes four types regarding the size of the defect, if it is a primary or recurrence parastomal hernia, and if there is simultaneous incisional hernia [11]. According to this, in our case, we had a patient with type III parastomal hernia, which means a primary parastomal hernia with a defect over 5 cm. The management of parastomal hernia is related to the clinical status of the patient; in the case, it is complicated with strangulation or perforation or obstruction where the patient must undergo an urgent surgery [8]. Carne et al. mentioned three types of surgical restoration of the hernia, which include mesh placement, change of stoma location, and local repair without however favorable results [8]. The European Hernia Society suggested the placement of a prophylactic mesh simultaneous to the creation of stoma to prevent the formation of parastomal hernia [2]. In this case, we followed a conservative plan of treatment in the patient like in another case of gastric parastomal hernia described by Waheed et al. [12]. We agree with the statement of the European Hernia Society that in patients with a parastomal hernia, the application of a hernia support belt may be useful in terms of reducing the risk of complications [2].

## Conclusions

Despite that parastomal hernia is a common complication after surgical procedures that lead to the formation of a stoma, the presence of the stomach in the hernia sac is rare and may result in complicated events that increase mortality and morbidity of a patient. We believe that it is important to highlight this rare clinical entity to reinforce further literature around it. The inclusion of patients with a stoma into a surveillance program may be useful regarding the early detection of parastomal hernia or other stoma-related complications.
